# Repeatability and degree of territorial aggression differs among urban and rural great tits (*Parus major*)

**DOI:** 10.1038/s41598-018-23463-7

**Published:** 2018-03-22

**Authors:** Samuel I. Hardman, Sarah Dalesman

**Affiliations:** 1 The Institute of Biological, Environmental and Rural Sciences, Aberystwyth University, Aberystwyth, SY23 3DA UK; 2 Communication and Social Behaviour Group, Max Planck Institute for Ornithology, 82319 Seewiesen, Germany

## Abstract

Animals in urban habitats face many novel selection pressures such as increased human population densities and human disturbance. This is predicted to favour bolder and more aggressive individuals together with greater flexibility in behaviour. Previous work has focussed primarily on studying these traits in captive birds and has shown increased aggression and reduced consistency between traits (behavioural syndromes) in birds from urban populations. However, personality (consistency within a behavioural trait) has not been well studied in the wild. Here we tested whether urban free-living male great tits show greater territorial aggression than rural counterparts. We also tested predictions that both behavioural syndromes and personality would show lower consistency in urban populations. We found that urban populations were more aggressive than rural populations and urban birds appeared to show lower levels of individual behavioural repeatability (personality) as predicted. However, we found no effect of urbanisation on behavioural syndromes (correlations between multiple behavioural traits). Our results indicate that urban environments may favour individuals which exhibit increased territorial aggression and greater within-trait flexibility which may be essential to success in holding urban territories. Determining how urban environments impact key fitness traits will be important in predicting how animals cope with ongoing urbanisation.

## Introduction

Individual differences in behaviour within species or populations are of increasing interest to behavioural and evolutionary ecologists^1–4^ and evidence that individuals behave consistently differently to one another across time, contexts and situations has now been found in a wide range of both vertebrate and invertebrate taxa including mammals^5^, birds^6^, insects^7^ and cnidarians^8^. Such consistent individual differences in behaviour have been referred to in various studies as temperaments^4^, coping styles^1^, behavioural types^5^ or animal personalities^9^.

In non-human animals high within individual repeatability has been shown for character traits such as aggression^7^, boldness^10^, willingness to explore new environments^11,12^, neophobia and activity level^12^. Furthermore, many species exhibit behavioural syndromes in which suites of independent behavioural traits are consistently positively correlated^2,3^. For example, aggression and boldness often correlate to form a behavioural syndrome such that more aggressive individuals are also bolder^2,3^. Here we refer to consistent individual differences in single behavioural traits as personalities and correlations between multiple behavioural traits as behavioural syndromes.

Personalities have a heritable component^6,13,14^ and their ubiquity across the animal kingdom implies either limits to adaptive behavioural plasticity^2,15^, that personalities arise during development^16^, or that consistent individual differences in behaviour may be strongly favoured by natural selection^17^. Given that variable environments would likely favour, rather than constrain, the evolution of behavioural plasticity, many studies have begun to focus on studying behavioural types from an evolutionary perspective^15^. Behavioural consistency in adulthood may have a genetic basis, but also be flexible, dependent on the developmental environment(e.g. food availability^18^; social environment^19^) or the current adult environment (e.g.^20^), or influenced by both genes and environment (e.g.^14^). Indeed, personalities have now been associated with key elements of evolutionary fitness such as survival^9^ and reproductive success^5,21^. Furthermore, individuals with different personality types have been shown to play different ecological roles (e.g. exploit different resources or ecological niches)^10,22^. If some personality types are better suited to dealing with certain challenges than others, personality could determine how successfully individuals are able to occupy a range of different environments with different selective pressures^23^. Therefore, the study of animal personalities in populations occupying changing or recently altered habitats may help to explain how animals cope with human-induced rapid environmental change (*sensu*^24^).

In the United Kingdom urban environments have been defined by the Office for National Statistics as areas of irreversibly developed land (buildings and glasshouses, asphalt, concrete and gardens) of at least 20 ha (20,000 m^2^) in size with a human population of at least 1500 people^25^. Animals inhabiting urban environments face novel selective pressures such as noise pollution^26^, habitat transformation and the introduction of non-native species^27^. This human-induced rapid environmental change^24^ is challenging the persistence of biological communities worldwide^27^. As a result of human activity animal populations are experiencing sharp declines in biodiversity and urban populations are becoming increasingly homogenous as the same few species dominate urban habitats across the globe^28^.

Urban environments also differ from rural environments in ways which may be advantageous to some species. For example, urban areas are typically warmer than rural areas^29^ and often provide access to anthropogenic food sources^30^ creating a more stable habitat over time. However, they may also experience high levels of human disturbance within the habitat^27–30^, high population densities compared to their rural counterparts^31,32^ and a paucity of natural food sources with which to provision their offspring^33^. A reduction in food resources may be a function of changes in habitat. For example, greater gaps between trees in urban parkland compared to rural woodland is thought to increase energetic expenditure when foraging for young, resulting in smaller brood sizes and lower body weight in urban great tits^34^. The type of trees available within the habitat can also affect adult foraging behaviour when feeding young in both great tits and blue tits, so planting preferences within urban habitats have the potential to negatively impact breeding performance^35^. This may explain why in urban bird populations adult survival, particularly over winter, is increased, but urban environments may nevertheless be detrimental to productivity^33^. Understanding the differences among urban and rural populations in species that successfully inhabit urban environments will help us to determine how species alter their behaviour in response to urbanisation. This will aid us in identifying the selective pressures the urban environment imposes on species and assist towards understanding why some species fail to successfully adapt to urbanisation.

Evidence that behaviour differs between urban and rural populations of the same species has been shown in many taxa including mammals, amphibians and birds (reviewed in^30^). In birds, possibly the best studied behavioural traits associated with urbanisation are increased boldness^36–42^ and aggression^30,38,39,43^. For example, both Evans *et al*.^38^ and Scales *et al*.^37^ found that song sparrows in urban environments responded more aggressively towards song playbacks simulating a territorial intruder than did rural birds. Furthermore, in both of these studies song sparrows in urban habitats exhibited increased boldness towards humans as they allowed humans to approach more closely before flying away than individuals in rural environments. Similarly, Møller^42^ found that in 44 European bird species urban populations had consistently shorter flight distances when approached by humans. Urban birds have also been shown to exhibit increased boldness when faced with novel food sources. Sol *et al*.^44^ found that common mynas (*Acridotheres tristis*) from urban habitats were quicker to approach novel food sources and solve foraging tasks than individuals from rural habitats^44^. Why urban birds should exhibit increased boldness and aggression in urban environments is not fully understood. However, Duckworth and Badyaev^45^ found that increased aggression in western bluebirds (*Sialia mexicana*) allowed this species to out-compete another species, the mountain bluebird (*Sialia currucoides*), and extend its range into new environments. Increased boldness in urban populations may be explained by habituation to urban conditions where levels of human disturbance is high in comparison to rural environments. Furthermore, a tendency to be more explorative may allow birds in urban environments to exploit the novel food resources these habitats provide^30,44^.

The great tit (*Parus major*) is a model species for studies of personality in animals and the existence of personality in this species is strongly supported by multiple laboratory-based studies (e.g.^6,9,15,46–49^). Recent work has also demonstrated that great tits taken from the wild and tested in captivity exhibit personality in territorial defence^50^, demonstrating elements of both heritability and plasticity on consistency among experimental plots varying in population density^14^. Great tits are a common passerine which occupies both urban and rural habitats across Europe. A recent study of behavioural differences in urban and rural great tits found that individuals in urban habitats exhibit higher rates of pecking and distress calling than rural birds when captured and handled^51^. This result may indicate the presence of a more “proactive” (more explorative, less neophobic) personality type in urban populations. Personality and behavioural syndromes in great tits have also been tested in several other studies^6,9,15,46–48^, but in these cases comparisons between rural vs. urban populations were carried out on birds taken from the wild into captivity and this may have altered the way individuals behaved. In another passerine species, the song sparrow (*Melospiza melodia*), two previous studies have tested for differences in behavioural syndromes (individual differences in between-trait covariance) in wild urban and rural populations^37,38^. In both of these studies, boldness and aggression were found to correlate to form a behavioural syndrome in rural, but not in urban, song sparrow populations suggesting behavioural syndromes may break down in urban environments. However, these studies did not assess personality in individual behavioural traits in urban and rural habitats. Our study will add to this work by providing the first assessment of animal personality (within-trait consistency) in free-living rural and urban birds.

The aim of the present study was to test for differences in behavioural consistency between populations of birds living in sparsely populated rural areas and in densely populated urban areas. Here we examined personality and behavioural syndromes in territorial aggression in free-living great tits in urban and rural sites in and around two medium sized cities in the UK with population sizes of 342,627 (Leicester) and 254,251 (Derby)^52^. Male great tits were exposed to playbacks of locally recorded great tit song simulating an intruder in their territory^50,53^. Aggressive responses were then recorded over two consecutive days and individual repeatability in these responses was used to determine personality. Co-variance among behaviours displayed was used to assess behavioural syndromes. In this study we aim to address the following questions: (1) Do great tits show consistent individual differences in individual behavioural traits (personality) in their natural habitats?; (2) Are there significant correlations between different behavioural responses in urban and rural populations which could indicate the existence of behavioural syndromes?; and (3) Do differences in urban and rural environments lead to differences in personality in urban and rural populations?

We aimed to answer these predictions using playback experiments to test for individual consistency and population-level differences in the strength of the responses of urban and rural birds to song playbacks. We predict that that great tits will show evidence of personality in the wild as found in previous long-term studies on nesting populations in the natural environment^14,50^. Furthermore, we hypothesise that environmental differences between urban and rural habitats, such as high levels of human disturbance^27–29^, noise pollution^26^ and increased competition from non-native species^27^, will lead to personality differences in individuals from urban and rural populations. Boldness and aggression are likely to form a behavioural syndrome as both of these behaviours have been shown to correlate in several other bird species^38,54,55^. Increased habitat stability has been shown to decrease the strength of covariance among traits^56^ and urban great tits showed reduced covariance in urban compared to rural habitats for exploration and neophobia^49^; therefore, we predicted that individuals from urban populations would show reduced covariance among behavioural traits related to territorial aggression compared to rural birds. Finally, as great tit populations occur at higher densities in urban than in rural habitats^31,32^ we predict that urban habitats may favour bolder individuals which are more willing to take risks and are more aggressive^37,38^. These traits may be useful when competing for limited food^31^ and territories^14^ and when defending smaller and more densely packed territories^14^ in which rival males are likely to encounter each other more often than in rural habitats.

## Results

### Repeatability

**Figure 1:**
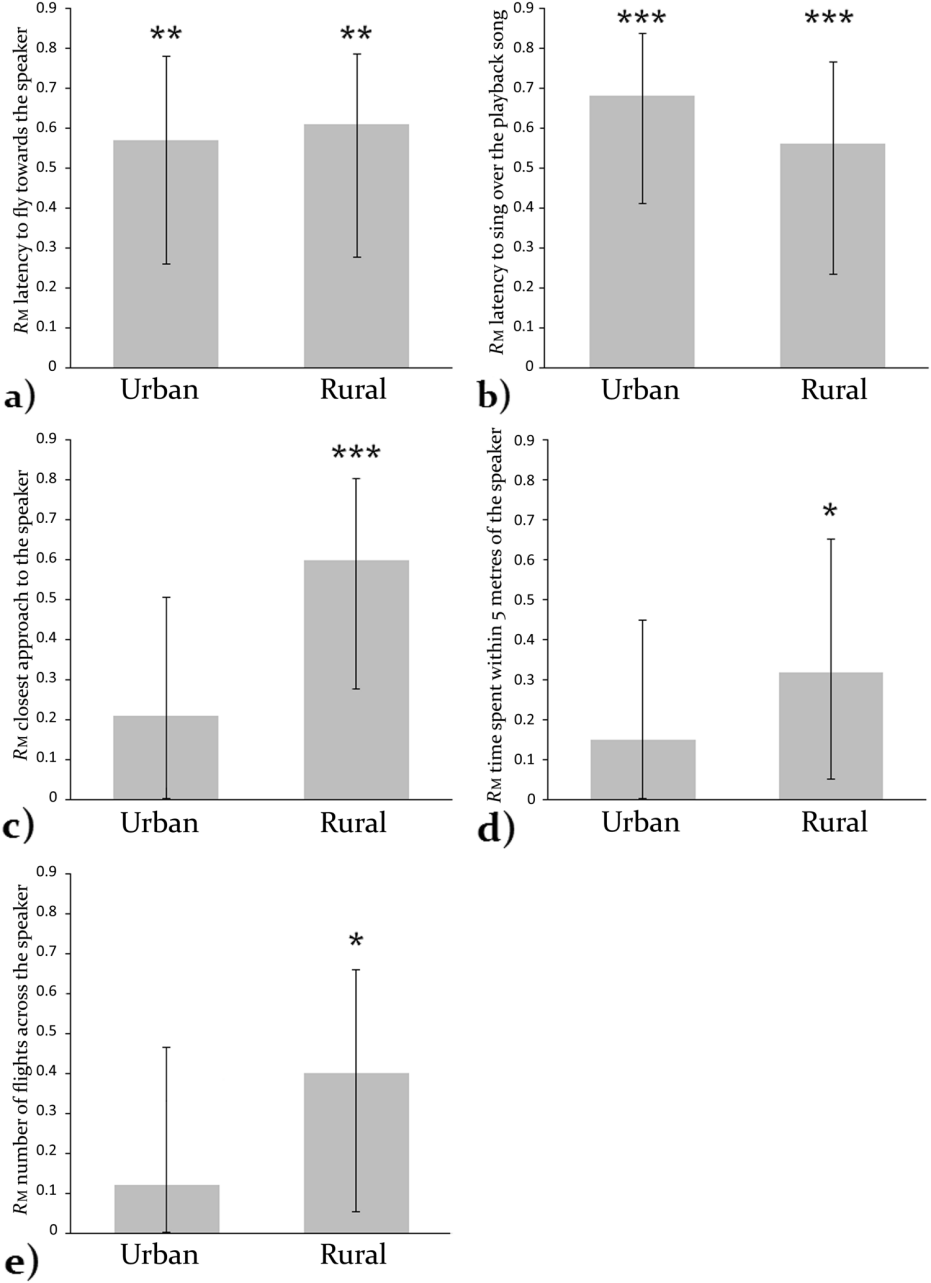
Repeatability scores for all five response behaviours for urban and rural great tits (±95% CI) for each of the following five behaviours: (**a**) Latency to fly towards the speaker, (**b**) Latency to overlap the playback song, (**c**) Closest approach to the speaker, (**d**) Time within five metres of the speaker, (**e**) Number of flights across the speaker. Repeatability is significant where the error bars do not reach zero. The overlapping error bars between urban and rural populations indicate no significant difference in repeatability between urban and rural birds for any behaviour. Significant repeatability after FDR correction is indicated by asterisks (*<0.05; **<0.01; ***<0.001).

For rural birds we found evidence of statistically significant repeatability for all five behaviours measured in response to song playbacks. In contrast, urban birds only exhibited significant repeatability in two behaviours (LatFly and LatSing) while the repeatability of the other three behaviours (Flights, Approach and Time5) was not significant (Table 1; Fig. 1). The low linear mixed-model based repeatability (*R*m) scores for urban birds for these three behaviours indicates high within-individual variance and low behavioural consistency in these traits. The overlapping 95% confidence intervals for urban and rural great tits for all five behaviours indicates that repeatability did not differ significantly between urban and rural populations for any of the five response behaviours. However, as the confidence intervals were large in all cases it is difficult to draw firm conclusions about the difference (or lack of) in repeatability between urban and rural populations.

**Table 1:** Linear mixed-model based repeatability scores (*R*m) for urban and rural great tits for all five response behaviours.

Behaviour	Habitat type	Rm	s.e.m.	95% CI (lower, upper)	P-value
LatFly	Urban	0.57	0.14	0.262, 0.780	0.001*
	Rural	0.61	0.13	0.278, 0.786	0.002*
LatSing	Urban	0.68	0.11	0.411, 0.836	<0.0001*
	Rural	0.56	0.14	0.234, 0.765	0.0006*
Approach	Urban	0.21	0.16	0, 0.506	0.136
	Rural	0.60	0.14	0.278, 0.804	<0.0001*
Time5	Urban	0.15	0.137	0, 0.451	0.195
	Rural	0.32	0.17	0.051, 0.654	0.016*
Flights	Urban	0.12	0.14	0, 0.464	0.28
	Rural	0.40	0.16	0.053, 0.658	0.018*

Scores range from zero to one where a score of zero indicates low repeatability and high within-individual variance and a score of one indicates high repeatability and low within individual variance. Significant results are indicated with an asterisk.

### Correlations between behaviours

For urban and rural great tits we found a significant negative correlation between 1) Approach and Flights and a significant positive correlation between Flights and Time5. These results show that birds which approached the speaker more closely at any point during playbacks (Approach) or spent longer within five metres of the speaker during playbacks (Time5) also performed more flights across the speaker during playbacks. A significant negative correlation between Approach and Time5 was also found for urban and rural great tits indicating that birds which approached the speaker closely at any point during the playback remained within five metres of the speaker for longer (Table 2).

**Table 2:** Partial Pearson correlations between different response behaviours for urban and rural great tits.

Behaviour	Habitat type	Partial correlation	t	df	Adjusted P-value
LatFly × Approach	Urban	0.39	2.08	24	0.13
	Rural	0.09	0.47	24	0.71
LatFly × LatSing	Urban	0.22	1.11	24	0.37
	Rural	0.13	0.65	24	0.61
LatFly × Time5	Urban	−0.36	−1.87	24	0.15
	Rural	−0.003	−0.012	24	0.99
LatFly × Flights	Urban	−0.33	−1.75	24	0.18
	Rural	−0.14	−0.70	24	0.61
Approach × LatSing	Urban	−0.23	−1.18	24	0.36
	Rural	−0.26	−1.29	24	0.32
Approach × Time5	Urban	−0.83	−7.23	24	<0.0001*
	Rural	−0.60	−3.69	24	0.005*
Approach × Flights	Urban	−0.75	−5.57	24	<0.0001*
	Rural	−0.58	−3.48	24	0.008*
LatSing × Flights	Urban	0.29	1.49	24	0.25
	Rural	0.30	1.53	24	0.25
LatSing × Time5	Urban	0.38	2.03	24	0.13
	Rural	0.03	0.17	24	0.90
Flights × Time5	Urban	0.80	6.73	24	<0.0001*
	Rural	0.50	2.80	24	0.03*

Significant results are indicated with an asterisk. P-values have been adjusted using the FDR correction method to account for multiple comparisons.

### Differences between urban and rural birds

**Figure 2:**
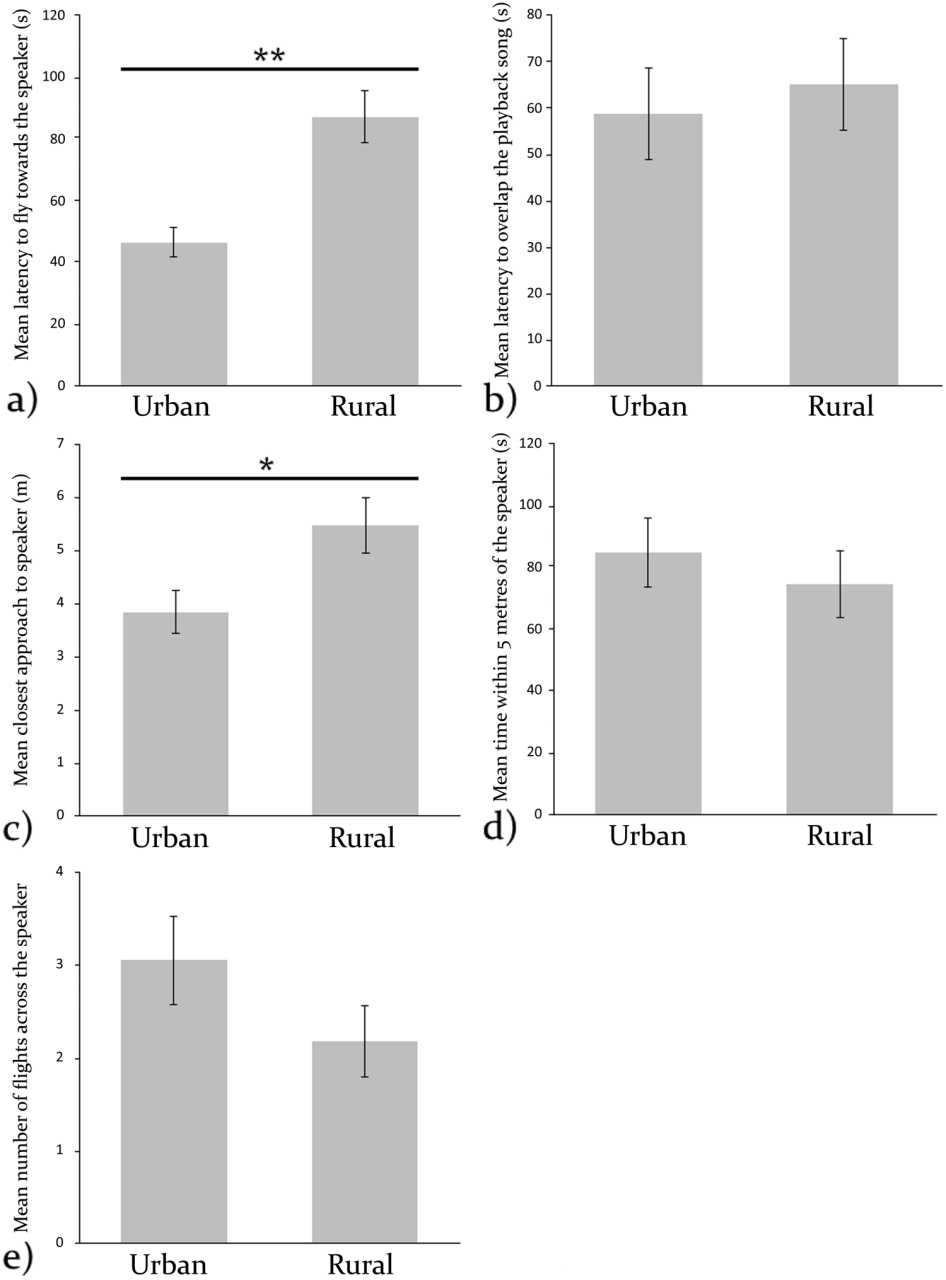
Bar charts showing the mean (± s.e.m.) for urban and rural great tits for each of the following five behaviours: (**a**) Latency to fly towards the speaker, (**b**) Latency to overlap the playback song, (**c**) Closest approach to the speaker, (**d**) Time within five metres of the speaker, (**e**) Number of flights across the speaker. Significant differences are indicated with asterisks (*<0.05; **<0.01).

Urban great tits were found to fly towards the speaker significantly faster than rural great tits (urban birds: mean 46.29 s, s.e.m. 4.81; rural birds: mean 81.63 s, s.e.m 7.57), and approached the speaker significantly closer than rural birds (urban birds: mean 3.83 m, s.e.m. 0.40; rural birds: mean 5.46 m, s.e.m. 0.53) in response to song playbacks (Table 3; Fig. 2).

**Table 3:** Differences in the responses of urban and rural great tits to song playbacks.

Behaviour	Log likelihood	deviance	χ^2^	df	P-value
LatFly	−255.36	510.72	10.53	1	0.001*
LatSing	−289.40	578.81	0.10	1	0.75
Approach	−117.94	235.89	5.43	1	0.02*
Time5	−326.57	653.14	0.87	1	0.35
Flights	−154.13	308.26	0.89	1	0.34

Significant differences are indicated with an asterisk.

## Discussion

Urban great tits demonstrated increased territorial aggression towards song playbacks compared to their rural counterparts. This finding confirms those of other researchers assessing behaviour in wild house sparrows, where territorial aggression was higher in urban compared to rural populations^37,38^. Other studies have found reduced aggression in urban birds, including finches^57^, house sparrows^58^ and great tits^59^. However, these studies were all carried out in captivity, and aggression was assessed in alternative contexts, either food aggression^57,58^ or response to handling following capture^59^. Differences between these studies and our own assessing relative aggression between urban and rural populations could be due to the context in which birds were displaying aggression. Aggression related to food acquisition and territorial defence may be under different selection pressures in the urban environment, favouring a reduction in food aggression, but increased territorial aggression. Differences could also arise due to the effects of captivity on these traits. Captivity has been shown to alter behavioural and physiological traits across a wide range of species e.g. reviews in^60,61^, including stress responses in birds^62^ which has also been linked to individual differences in territorial aggression^63^. In captivity, the conditions in which males are maintained can also affect aggressive behaviour^64^. Therefore, it is entirely feasible that testing birds in captivity also alters individual displays of aggressive behaviour compared to their behaviour in the wild. Past studies have shown that it is possible to test aggressive behaviours in wild birds in their natural habitats using song playbacks^65,66^ or model predators^66^ to induce aggressive responses. We suggest that future work should assess the same traits in both captive and wild birds to determine whether these differences are due to discrete effects of urbanisation on individual traits or an artefact of context (laboratory vs. wild) in which they are tested.

Rural great tits exhibited significant repeatability in all five behavioural responses measured, indicating individual consistency over time in traits related to territorial aggression in rural populations. In contrast, significant repeatability was found for only two of the five behaviours measured in urban great tits, namely their latency to fly towards the speaker and their latency to overlap the playback song, whilst other behavioural traits related to territorial aggression showed no individual consistency over time. Based on previous work it was not surprising that great tits demonstrated consistency within traits^6,9,14,15,48–50^. However, while past studies have identified differences in behavioural syndromes (consistent individual differences in between-trait covariance) in urban and rural passerine species^37,38,49,67^, our study indicates that animal personality (within-trait covariance) also differs between urban and rural populations of wild and free-living animals in natural habitats.

Despite repeatability being significant across all traits in rural birds and only two traits showing repeatability in urban birds, we did not find a significant difference in repeatability between urban and rural populations. This is likely the result of the large amount of among-individual variability in repeatability of traits within populations. Repeatability depends on both among-individual differences and within-individual consistency; however, consistency is likely to be context specific^68^, and conditions may fluctuate in the field. As the measures taken here were carried out on separate days individual birds may have experienced different conditions from day to day, for example territorial intruders, therefore influencing measures of repeatability which tends to decrease with increased duration between measures^69^. If population density is higher in urban environments this type of disturbance may occur more frequently, and therefore explain the reduced consistency in behaviour over time in urban populations. This could be addressed by measuring behaviour in the laboratory under constant conditions (as done in many studies), increasing the probability of detecting personality; however, as already mentioned this may also alter natural behaviours. Further work is needed to confirm whether this trend for decreased repeatability in urban great tits is a product of increased disturbance between measurements or maintained under constant conditions indicating selection for increased flexibility in behaviour in urban habitats.

Our results contrast with a study on hand-reared Eurasian blackbirds (*Turdus merula*) which found that individuals originating from parents in urban habitats appeared to show higher repeatability scores for neophilic and neophobic behaviours than rural blackbirds did^70^. However, neophobia and neophilia are likely to be under different selection pressures to aggressive displays, and rearing in common garden conditions will exclude environmental effects on behaviour of the adults. Higher population densities in urban environments^31,32^ may require greater individual flexibility in territorial aggression as well as higher overall aggression. This is supported by the results here which show that urban great tits are on average more aggressive than their rural counterparts. During playback experiments urban birds flew towards the speaker 35.34 seconds more quickly on average than rural birds and approached the speaker an average of 1.63 metres closer. While this result may alternatively be explained by increased neophilia in response to a novel object (the playback speaker) in urban birds, neither urban nor rural birds showed any response to the speaker until playbacks began making this possibility unlikely. Higher population densities of birds, including great tits, in urban environments^31,32^ and more densely packed territories mean that urban males are likely to encounter intruders on a more regular basis than in rural environments which may lead to increased aggressive behaviour^32^. Indeed, high population density has been linked to increased territorial aggression in previous work on great tits^14^. Therefore, urban males will potentially have to demonstrate greater plasticity in aggressive behaviours in order to maintain their territories whilst at the same time reducing the costs associated with aggressive displays when they are not required.

In addition to consistency in single traits across time we also found correlations between different behavioural responses to playback songs which may be indicative of a behavioural syndrome in this species. In both urban and rural populations the time individuals spent within five metres of the speaker and their closest approach were correlated with the number of flights birds made across the speaker during playbacks. Both approaching closely and flying across the speaker (simulating an intruder) are known aggressive behaviours in great tits and usually occur when a conflict has escalated and using song as a deterrent has not been effective at driving an intruder out of a territory^71^. Furthermore, as well as being a known aggressive behaviour, approaching an intruder closely could also be considered a bold behaviour as it can potentially lead to physical aggression and injury^11^ and is therefore risky. Aggression with neighbouring conspecifics may also be costly as individuals engaged in fights are unlikely to be vigilant against predators and other potential threats. The negative correlation between the closest approach individuals made to the speaker or the time they spent within five metres of the speaker and the number of flights they performed across the speaker may therefore reflect evidence in the wild of the behavioural syndrome including boldness and aggression first identified in great tits by Verbeek *et al*.^11^.

Previous studies have indicated that boldness and aggression positively correlate within individuals to form a behavioural syndrome in great tits^11^ and many other species (e.g.^37,38,72,73^). For example, song sparrows which are bolder towards humans are also more aggressive when defending their territories^38^. However, a later study found that while boldness and aggression are positively correlated in this species as a whole, in urban populations this behavioural syndrome breaks down and boldness and aggression vary independently of one another^37^. Similarly, Bókony e*t al*.^74^ found that behavioural syndromes present in rural populations of house sparrows (*Passer domesticus*) break down in urban populations. Evidence that behavioural syndromes break down in urban populations has also been found in great tits. In this species exploration and neophobia have been shown to correlate to form a syndrome in forest birds but not in urban birds^49^. These results suggest that urbanisation or human disturbance directly affects the strength of correlations between behavioural traits. Possibly because lower predation in urban habitats allows shyer, or less bold, individuals to behave more flexibly and express higher boldness, thus leading to the breakdown of behavioural syndromes^37,75^.

In contrast to these studies we found no evidence that behavioural syndromes differed between urban and rural populations. Past studies have suggested that behavioural syndromes break down in high quality habitats^76,77^ with lower predation risk^75^. However, in fish exposure to predation risk has been found to both enhance^76^ and break down^78^ correlations among behavioural traits, and increased habitat stability resulted in a reduction in trait covariance in gastropods^56^, therefore the picture is far from clear. It is possible that the urban sites used in the present study did not differ substantially from rural habitats in quality or in predation risk and this would explain why no difference in behavioural syndromes was found between urban and rural populations. It may also be that the measures we took were all elements of ‘aggression’ and therefore could not be considered individual traits that are subject to differing selection pressures. Traits related to territorial aggression may be under strong selection pressure to be maintained in the urban environment where high population density selects for effective aggressive displays^14^, and if alternate behavioural traits unrelated to aggression were assessed a breakdown in covariance may be identified. Furthermore, urban habitats themselves may differ from each other in ways which affect the behaviour of birds. Further studies in a broader range of urban habitats are therefore required to fully understand the relationship between urbanisation and behavioural syndromes.

This study demonstrates that differences in populations of the same species can emerge as a result of urbanisation. It is unlikely that habitat differences unrelated to urbanisation (i.e. elevation, local flora & fauna etc.) can explain the differences in behaviour observed in urban and rural populations as all sites were located in the same geographic region of the UK and were chosen to be similar in all properties except for degree of urbanisation. Although previous studies have shown evidence of differences in behavioural syndromes between urban and rural animal populations^37,38,67^ this is the first study to show that urbanisation can affect behavioural consistency in individual traits measured in the wild. Past studies have shown that human activities can strongly affect animal behaviour (e.g.^37,38,42,67,79^). Urbanisation may therefore act as a selective force driving the evolution of behavioural and phenotypic differences between urban and rural populations^42,80,81^. As urban areas continue to grow, the impact on animal populations is likely to increase and may influence the evolution of urban populations in unpredictable ways. Although past work has shown that selection may play a key role in adaptation to urban environments^49,70,82,83^, developmental plasticity and epigenetics may also be important but their roles in this context are not well understood. For this reason, future studies aiming to determine the mechanisms driving the differences between urban and rural populations of birds would be particularly useful and may allow us to predict which species will be able to adapt to an increasingly urban world.

## Methods

### Study sites

**Figure 3:**
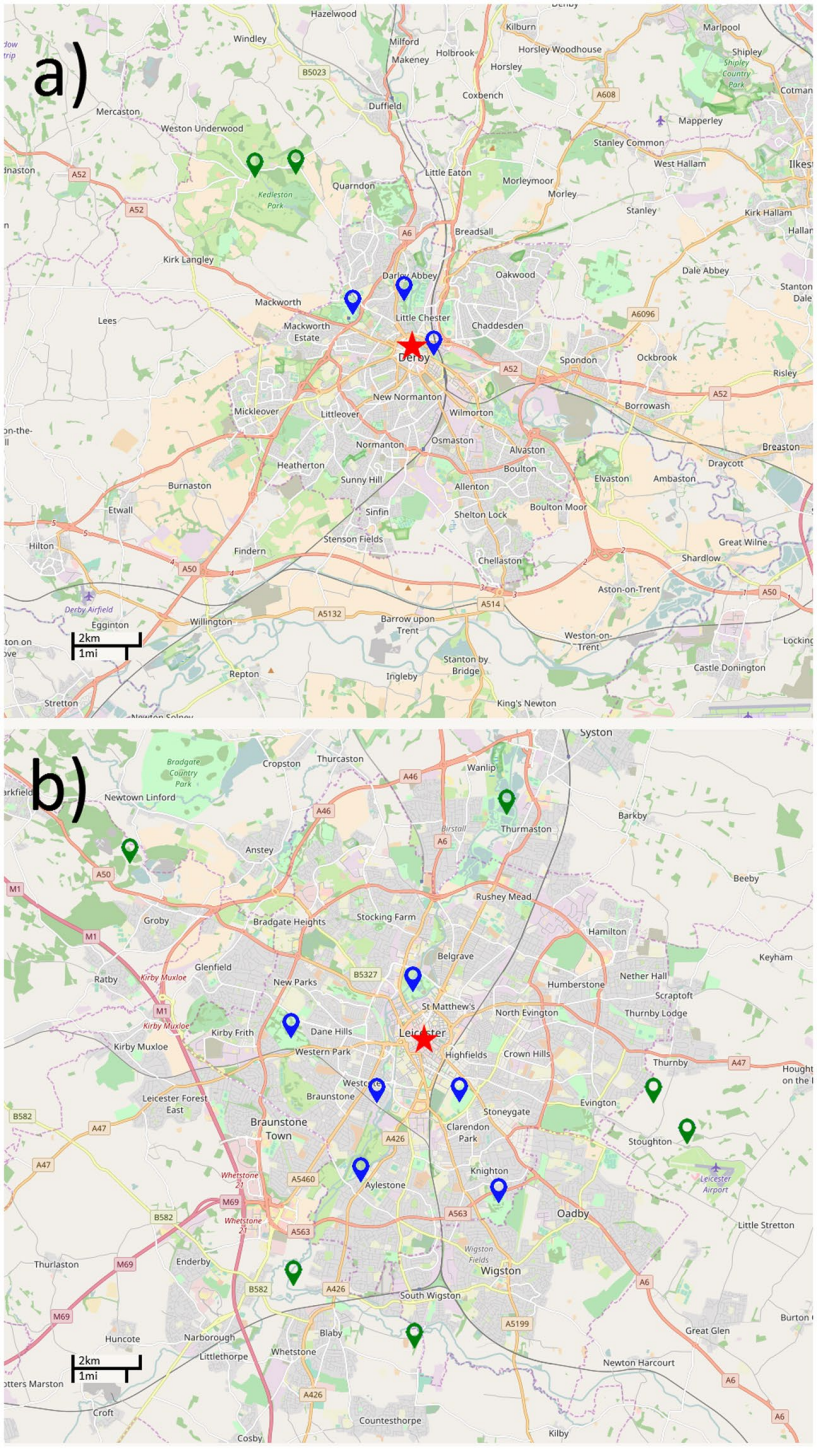
Maps showing the location of playback experiments carried out in Derby (map a) and Leicester (map b). Blue markers show urban sites and green markers show rural sites. Multiple experiments were carried out on different great tits at each site. The red stars mark the position of the city centres. Areas on the map with a dark grey background are urbanised. In total 54 playback tests were carried out on urban great tits (Derby *n* = 24, Leicester *n* = 30) and 54 tests were carried out on rural great tits (Derby *n* = 20, Leicester *n* = 34). Rural sites were a mean distance of 6.36 km (±0.26 s.e.m.) from the city centre while urban sites were a mean distance of 2.18 km (±0.40 s.e.m.) from the city centre. The mean distance between rural sites and the nearest urban site was 4.42 (±0.33 s.e.m.). The mean distance between rural sites was 5.29 km (±1.61 s.e.m.). The mean distance between urban sites was 2.15 km (±0.25 s.e.m). Our GPS points (Map data copyrighted OpenStreetMap contributors and available from https://www.openstreetmap.org, CC-BY-SA).

Playback experiments were conducted in urban and rural sites (as defined by the United Kingdom Office for National Statistics^25^) in and around Leicester (population 329,839) and Derby (population 248,752) in the United Kingdom between 9^th^ March 2015 and the 24^th^ April 2015. Urban sites consisted of small parks close to the city centres sparsely populated with trees and containing areas of mown grass, flower beds, and ornamental shrubs and surrounded by high-density urban developments such as housing, industrial and commercial buildings and major roads. Rural sites were located outside of the cities and consisted of deciduous woodland surrounded on all sides by open farmland. In both urban and rural sites great tit territories were centred around at least one tree (containing the nest) and were around 60 metres in diameter^84^. A total of 9 urban sites (6 in Leicester, 3 in Derby) and 8 rural sites (6 in Leicester, 2 in Derby) were visited during this study. Rural sites were a mean distance of 6.36 km (±0.26) from the nearest city centre and 2.77 km (±1.3) from the next nearest rural site while urban sites were within city boundaries and a mean distance of 2.18 km (±0.40) from the nearest city centre and 1.5 km (±0.33) from the next nearest urban site. The minimum distance between an urban site and a rural site was 2.45 km (Fig. 3 ^85^).Given that great tits typically disperse less than one kilometre from the site at which they hatched^46,86^ these distances were sufficient to ensure that the urban and rural populations sampled in the study did not overlap and individuals sampled in urban sites were very unlikely to have moved there from rural sites and vice versa.

### Playback songs

For each playback experiment a new playback song was recorded locally in each study site (at least 250 m away from the bird receiving the playback) as previous studies have shown that great tits respond more strongly to song types which are familiar to them^65^.In each urban and rural site the songs of local territorial males were recorded from within 10 metres using a Sennheiser (Wedermark, Lower Saxony, Germany) ME67 unidirectional microphone attached to a Tascam (TEAC Corporation, Montebello, CA, USA) DR-680 digital recorder with a sampling rate of 44,100 Hz. The recordings were prepared for use in playback experiments using Avisoft SAS lab lite v. 5.2.07 (Avisoft bioacoustics, Berlin, Germany) with the fast Fourier transform length set at 256 with 50 percent frame in a Hamming window which gave a resolution of 172 Hz and 2.9 ms.

Before use in playback experiments song recordings were filtered using the high-pass filter function in Avisoft to remove excess low-frequency noise. Cut-off frequencies for songs were decided after visual inspection of each spectrogram to avoid unintentionally removing part of the song. From each filtered playback recording two notes from the middle of a strophe were clipped from the spectrogram and repeated four times to produce an eight-note strophe that was looped for five minutes to create each playback song. A short pause was left between strophes equal to the length of the pause between strophes in each original recording.

### Playback experiments

Within each study site territorial males were identified by walking through a suitable habitat until a bird was found. The extent of their territories were then determined by 30 minutes of observation. To be classified as a male’s territory the male remained within an area for the full 30 min observation period. Once a bird’s territory had been identified a FoxPro wildfire 2 speaker (FoxPro Inc., Lewistown, PA, USA) mounted on a tripod 1.5 metres from the ground was then placed in the centre of the territory and five minutes of song playback began once the bird had come within 25 m of the speaker (following the procedure used by^65^). No approach to the speaker was observed prior to playback commencing (pers. obs.), and previous work has shown that great tits do not respond to the speaker being placed in their territory with aggressive behaviour^87^. Song playbacks were broadcast at 69 dB (A-weighted; reference level 20 μPa) measured ten metres from the front of the speaker using a CEM DT-805 sound level meter. During each song playback the behaviour of the focal bird was observed (by S.I.H) and five response behaviours were recorded continuously by the same observer. These were: (1) the latency to fly towards the speaker (LatFly); (2) the latency to sing over the playback song (LatSing); (3) the total number of flights across the speaker (Flights); (4) the closest approach to the speaker during the playback (Approach); and (5) the total time spent within five metres of the playback speaker (Time5)^65^. Latency to fly towards the speaker and to sing over the speaker were measured in seconds using a stopwatch. Flights across the speaker were counted when the bird flew from one side of the speaker to the other crossing directly above the speaker. The proximity of the bird to the speaker was measured in metres using a tape measure which was laid out prior to each experiment. A 105 CEM DT-805 sound level meter (A-weighted; reference level 20 μPa) was used to standardise the sound pressure level of all song playbacks to 69 dB at 10 metres from the speaker^84^. Experiments took place between 10.00 am and 3.00 pm each day and one site was visited per day. The number of experiments carried out each day varied from one to six. Experiments were only carried out during dry and calm weather. As noise levels are known to be lower in urban habitats on weekends^88^ urban sites were only visited during weekdays (Monday-Friday). As noise levels in rural habitats do not differ across the week these sites were visited on both weekdays and weekends. Urban and rural sites were visited in a random order.

### Measuring repeatability

To measure individual repeatability in response behaviours identical playback experiments were conducted once per day over two days. For each bird playbacks were timed to occur at the same time of day (+/− 1 hour) for both repeats. For each playback experiment a new song was recorded on the same day as the playback test so that no playback song was used more than once. As male great tits defend small territories during the breeding season which they rarely leave^89^, we worked on the assumption that we were able to relocate the same individuals for each of the two playback repeats and are confident that this was the case.

### Statistical analyses

All statistical analyses were carried out using R v. 3.0.2^90^. Prior to analyses the data were square root transformed which provided normality of residual variances.

### Testing repeatability of responses to playbacks

To test the effect of habitat type (urban or rural) on the repeatability of the five response behaviours we calculated linear mixed-model based repeatability (*R*m) and 95% confidence intervals for all five response behaviours in urban and rural populations using the rptR package (version 0.6.405^91^). We used Gaussian models and included the ID of the birds’ territory nested within the location (Leicester or Derby) as random effects. Population level repeatability in a trait can be expressed as:

1 $$R{\rm{M}}=\frac{between\,individual\,variance}{(between\,individual\,variance+residual\,variance)}$$

The index of repeatability ranges from 0 (low repeatability and high within-individual variance) to 1 (high repeatability and low within individual variance).

In total 27 urban (Derby *n* = 12, Leicester *n* = 15) and 27 rural (Derby *n* = 10, Leicester *n* = 17) birds were tested on two separate occasions each giving a total of 108 playback experiments. On three occasions birds did not sing back to the playback song (LatSing). For this behaviour these birds were given the maximum score of 300 seconds (five minutes) equal to the full duration of the playback.

### Testing for correlations between different response behaviours

To test for correlations between pairs of different response behaviours in urban and rural birds we estimated partial correlations which test for association between two variables while controlling for one or more other variables. Partial correlations were estimated using the pcor package^92^. To control for multiple comparisons, we adjusted the P-values using the false discovery rate (FDR) method^93^.

### Testing for differences between urban and rural birds

Differences in the responses of urban and rural birds to song playbacks were tested for each response behaviour using Gaussian generalised linear mixed effect models (GLMMs) using the package lme4^94^. We set the behaviour as the response variable, the habitat type (urban or rural) as a fixed effect and the ID and location (Leicester or Derby) of each bird as random intercepts. P-values were calculated by comparing models including habitat type as a fixed effect to null models using analysis of deviance tests.

### Data accessibility

Data are available as supplementary material.

## Electronic supplementary material

Supplementary Dataset 1
